# The effect of orthodontic treatment on smile attractiveness: a systematic review

**DOI:** 10.1186/s40510-023-00456-5

**Published:** 2023-02-06

**Authors:** G. Coppola, I. Christopoulou, N. Gkantidis, C. Verna, N. Pandis, G. Kanavakis

**Affiliations:** 1 Department of Pediatric Oral Health and Orthodontics, University Center for Dental Medicine Basel (UZB), University of Basel, Mattenstrasse 40, 4058 Basel, Switzerland; 2 Department of Orthodontics, School of Dentistry, National and Kapodistrian University of Athens, Athens, Greece; 3 Department of Orthodontics and Dentofacial Orthopedics, University of Bern, Bern, Switzerland; 4 Private Practice, Corfu, Greece; 5 Department of Orthodontics and Dentofacial Orthopedics, Tufts University School of Dental Medicine, Boston, MA USA

## Abstract

**Background:**

Smile attractiveness is a primary factor for patients to seek orthodontic treatment, however, there is yet no systematic evaluation of this topic in the literature.

**Objectives:**

To assess the current evidence on the effect of orthodontic treatment on smile attractiveness.

**Search methods:**

Seven electronic databases (MEDLINE, Cochrane Library, Virtual Health Library, SCOPUS, Web of Science, Google Scholar and Embase) were searched on 14 September 2022.

**Selection criteria:**

Studies evaluating smile attractiveness before and after orthodontic treatment or only after completion of orthodontic treatment.

**Data collection and analysis:**

Extracted data included study design and setting, sample size and demographics, malocclusion type, treatment modality and method for outcome assessment. Risk of bias was assessed with the ROBINS-I tool for non-randomised studies. Random-effects meta-analyses of mean differences and their 95% confidence intervals (CIs) were planned a priori.

**Methods:**

After elimination of duplicate studies, data extraction and risk of bias assessment according to the Cochrane guidelines, an evaluation of the overall evidence was performed. The included studies were evaluated based on the characteristics of their study and control groups and based on their main research question. Also, all outcome measures were standardized into a common assessment scale (0–100), in order to obtain more easily interpretable results.

**Results:**

Ten studies were included in this review, nine of which were assessed as being at serious risk of bias and one at moderate risk of bias. The large heterogeneity between the included studies did not allow for a meta-analysis. Orthodontic treatment has a moderately positive effect on smile attractiveness. When compared to no treatment, orthodontic treatment with premolar extractions improves smile attractiveness by 22%. Also, surgical correction of Class III cases increases smile attractiveness by 7.5% more than camouflage treatment. No other significant differences were shown between different types of treatment.

**Conclusion:**

Based on the available data, orthodontic treatment seems to moderately improve the attractiveness of the smile. There is significant bias in the current literature assessing the effect of orthodontics on smile attractiveness; therefore, the results cannot be accepted with certainty.

**Supplementary Information:**

The online version contains supplementary material available at 10.1186/s40510-023-00456-5.

## Background

Nonverbal communication is the most immediate and noticeable method of expression. Humans often show their emotions, consciously or non-consciously, through nonverbal channels, including facial expressions and gestures [1]. Communication between peers depends on how the signal is sent and how it is interpreted [1, 2]; thus, during a conversation, the attention of all involved parties is largely focused on the face and its expressions [3]. Smiling is the most common facial expression. A smiling face conveys happiness, politeness, self-confidence and therefore kindles positive social reactions [4]. However, depending on the occasion, smiling can also be a way to hide discomfort [5], create deception, or display dominance [6, 7]. A smiling face is viewed as more attractive compared to a non-smiling one and, at a neuronal level, its image elicits an increased response by the medial orbitofrontal cortex [8].

Due to the dominant role of smiling in everyday life [9, 10], there is an increasing demand for medical and dental treatments that improve smile and facial appearance, including orthodontic treatment. For most patients, an improvement in facial and smile attractiveness are the main motivating factors for receiving orthodontic treatment [11, 12].

In orthodontics, facial and smile esthetics are largely taken into consideration during the diagnosis and treatment planning and have been the focus of extensive orthodontic literature. In addition to applying the basic macro- and micro-esthetic principles of a pleasing smile in their diagnosis [13, 14], orthodontists are interested in evaluating the potential effect of tooth movement on smile and facial esthetics. The antero-posterior and vertical relationships of the anterior dentition, for example, have a significant effect on the soft tissue profile, particularly on the position of the upper lip [15, 16]. Therefore, it is essential to consider the potential impact of upper incisor retraction on lip posture and subsequently on lip esthetics [17–19].

Despite the general consensus that orthodontic treatment improves smile esthetics, there is little understanding of the actual impact that it has on the attractiveness of the smile. The terms “esthetics” and “attractiveness” are commonly used interchangeably in orthodontics, despite that they do not provide identical information. The perception of an esthetic smile is a cognitive, and therefore conscious process, which is influenced by certain “rules” for beauty, symmetry and facial harmony. The perception of an attractive smile, on the contrary, is a perceptual, neuronally more complex process, which is largely subjective [20–22]. For example, a more esthetically pleasing smile may be less attractive than a less esthetic, but more natural smile. Current evidence regarding the effect of orthodontic treatment on facial attractiveness demonstrates that individuals who have received treatment are perceived as 9% more attractive compared to untreated individuals; nevertheless, the quality of the available evidence is considered weak [23]. Furthermore, orthodontics is more likely to have a more significant effect on the smile than the entire face. However, the available information regarding the effect of orthodontics on smile attractiveness has yet not been reviewed and assessed systematically. Therefore, the purpose of the present systematic review was to evaluate whether orthodontic treatment has an effect on perceived smile attractiveness and to quantify the strength of this effect.

## Materials and methods

### Protocol registration and reporting

The present review was based on a specific protocol, developed and piloted following the guidelines outlined in the preferred reporting items for systematic review and meta-analysis protocols (PRISMA-P statement) [24] and has been registered at PROSPERO database (CRD42022331370) as of May, 2022. In addition, the conduct and reporting followed the Cochrane Handbook for Systematic Reviews of Interventions and the PRISMA statement, respectively [25, 26]. The review methods were established a priori and there were no consequent deviations to the initial research protocol. The design of the study and the construction of the study question were performed using the PICO model, as displayed in Table 1. The primary review question was whether orthodontic treatment has an effect on smile attractiveness compared to no orthodontic treatment. In addition, various types of orthodontic treatment were also compared in terms of their impact on smile attractiveness.

**Table 1:** Study inclusion and exclusion criteria (PICOS)

Field	Inclusion	Exclusion
Patients	Patients of any age, sex, ethnicity, and malocclusion	- Animal studies - In vitro studies - Patients with craniofacial syndromes or abnormalities
Intervention (exposure)	Phase I or Phase II orthodontic treatment with any type of fixed or removable appliance	Patient not receiving orthodontic treatment
Comparison	A. ortho-Tx vs no-Tx (No-Tx comprising data of other not treated patients) B. ortho-Tx vs ortho Tx C. pre ortho tx vs post ortho Tx	- Simulation of orthodontic treatment using software - Does not test the effect of orthodontic treatment - Tests only the effect of orthognathic surgery
Outcome	Qualitative and quantitative analysis of facial attractiveness as measured through standardized questionnaires and validated scales	- No clear mention of rating method - Rating of esthetics or facial esthetics based on orthodontic objective criteria
Study design	Randomized controlled trials or non-randomized, prospective or retrospective, cohort studies and observational, exploratory studies will be included	- Case reports, Systematic reviews, Meta-analyses, Reviews

### Literature search strategy

**Fig. 1:**
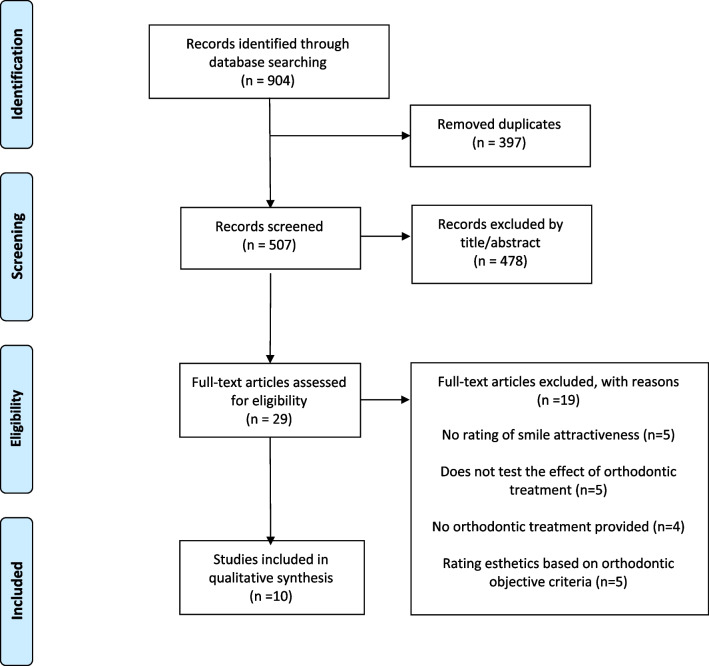
Flow diagram for the selection of studies according to PRISMA. (Diagram from: Moher D, Liberati A, Tetzlaff J, Altman DG, The PRISMA Group (2009). Preferred Reporting Items for Systematic Reviews and Meta-Analyses: The PRISMA Statement. PLoS Med 6(7): e1000097. https://doi.org/10.1371/journal.pmed1000097)

Seven electronic databases (Cochrane Library, Embase, Google Scholar, Medline, Scopus, Virtual Health Library, Web of Science) were searched from inception until September 2022 by two independent examiners (IC and GC). The flow chart for the various steps in the search is presented in Fig. 1. Detailed search strategies for each database are listed in Additional file 1: Table S1. The search was carried out without limitations with regard to language, status and year of publication. Grey literature was sought through openger.eu and greylit.org. The reference lists of the included studies were also hand-searched for additional relevant studies.

## Study selection

### Eligibility criteria

Randomized and non-randomized controlled trials, cohort studies and case–control studies, whether prospective or retrospective, were included. Case reports, systematic reviews, meta-analyses, and reviews were excluded. Studies involving phantoms, reference models, dry skulls, and dental casts were also excluded.

Patients of any age, sex, ethnicity, and malocclusion receiving phase I or phase II orthodontic treatment with any type of fixed or removable appliances or a combination of orthodontic and surgical treatment were eligible for inclusion. Studies using computer software to simulate orthodontic treatment, or studies including patients with craniofacial syndromes or abnormalities and cleft palate patients were excluded. Studies examining the effect of orthognathic surgery in isolation were excluded.

Moreover, a study was considered eligible if it reported a qualitative and quantitative analysis of smile attractiveness at all available time-points, measured through standardized questionnaires and validated scales. If the rating method or the used validated scale were not mentioned, or if only facial attractiveness was evaluated, the study was not considered eligible for inclusion. The selection criteria leading to the included studies are listed in Table 1. Studies excluded after full text reading, as well as the reasons for exclusion are listed in Additional file 2: Table S2.

Despite the significantly lesser likelihood of randomized studies to be affected by risk of bias compared to non-randomized studies, there are ethical considerations in conducting randomized studies including patients in need of treatment. Furthermore, it is not questionable whether patients with severe malocclusions will agree to participate in a randomized study and postpone a correction for which they have seeked orthodontic care. These limitations were acknowledged prior to conducting this review, and therefore both types of studies were included.

### Data extraction

Two authors (IC and GC) assessed the titles and the abstracts of the retrieved records for inclusion independently and in duplicate. They were not blinded to the identity of the authors, their institution, or the results of the research. Subsequently, they obtained and assessed, again independently, the full report of records considered by either reviewer to meet the inclusion criteria. Disagreements were resolved by discussion or consultation with the last author (GK).

Search results were imported into Endnote (Clarivate Analytics, PA, USA) for further selection. The same authors who search the databases also performed data extraction independently and in duplicate, and any disagreements were again resolved by discussion or consultation with the last author (GK).

Data on study characteristics included the study ID, study design, setting, country, university, patient number, sex distribution, age, ethnicity, type of treatment and method for rating smile attractiveness (type of raters, studied variable and evaluation tool). Numerical data of outcome measurements included means and standard deviations.

### Risk of bias assessment

For randomized studies, the RoB 2 tool [27] was used for the risk of bias assessment (GC and IC). For the non-randomized studies, the ROBINS-I tool [28] was used for the same purpose. Any disagreements were resolved by discussion or consultation with the last author (GK).

### Data synthesis

The outcome of interest was smile attractiveness assessed by panels of raters. The effect of various types of orthodontic treatment or a combination of orthodontic-surgical treatment on smile attractiveness was examined in the included studies through comparisons between different treatment methods or between treated and untreated control groups.

When evaluating the methodological heterogeneity of the included studies, it was determined that there was large diversity between the studies, in regard to differences in clinical characteristics of the patients’ sample, differences in treatment methods, type of clinical study (retrospective-prospective, randomized-non-randomized), study design and outcome assessment. Therefore, a meta-analysis of all data was not possible. Nevertheless, the following steps were taken to perform a synthesis of the individual results. The included studies and their individual groups (where applicable) were grouped according to their research question and the applied intervention. In addition, all rating scales used to evaluate smile attractiveness were adjusted into a 0–100 scale to provide comparable and more easily interpretable results.

## Results

### Study selection

The initial literature search generated 904 studies. After screening for doubles and applying the inclusion criteria to titles and abstracts, 29 articles were found eligible for full-text assessment. 19 of these 29 studies were excluded for various reasons, which are listed in the flow diagram (Fig. 1) and are also provided in detail in Additional file 1: Table S2. As a result of the above process, a total of 10 studies were included in this systematic review (Table 2).

**Table 2:** Characteristics of included studies

No	Study ID	Design; Setting; Country	Patient number (M/F) *; Type of malocclusion; Type of treatment (tx)	Age	Method for rating facial attractiveness		
					Rated image	Raters (M/F)	Evaluation tool
1	Almutairi et al., 2015	Observational; University and private clinics; Saudi Arabia	14 (0/14); Class II malocclusion/ Bimaxillary protrusion Tx_1_: 7; 4Ex + FA C: 7; No tx	Adults ≥ 16yrs	Smile photo (frontal and ¾)	50 laypeople (25/25) 50 general dentists (25/25) 50 orthodontists (25/25)	100-point scale
2	Havens et al., 2010	Retrospective; University; USA	48 (0/48); Not specified T0: 48 pre-tx T1: 48 post-tx	13.0–17.6 yrs	Smile photo and Smiling face photo	20 laypersons 20 orthodontists	8-point scale
3	Hulsey et al., 1970	Observational; Japan	40 (20/20) Not specified Tx: 20 Tx C: 20 No Tx	15–25 yrs	Smile photo	20 laypersons (10/10)	5-point scale
4	Janson et al., 2014	Retrospective; University; Brazil	66 (22/44); Class II division 1 Tx_1_: 23; 1Ex + FA Tx_2_: 23; 4Ex + FA Tx_3_: 20; 3Ex + FA	Group 1: 24.04 (4.97) Group 2: 25.40 (6.70) Group 3: 21.63 (5.27)	Smile photo	46 laypeople (18/28) 70 orthodontists (47/23)	10-point scale
5	Kumar et al., 2016	Retrospective; University; India	72 (N/A); Not specified T0: 72 pre-tx T1: 72 post-tx	Not specified	Smile photo	6 laypeople (3/3) 6 general dentists (3/3) 6 orthodontists (3/3)	10-point scale
6	Meyer et al., 2014	Retrospective; Dental Hospital; Australia	57 (24/33); Class II malocclusion Tx_1_: 30; 4Ex + FA Tx_2_: 27; Non-Ex + FA	Pre-treatment mean age: 14.87 (2.99)	Smiling face photo	20 laypeople (10/10) 20 general dentists (10/10) 20 orthodontists (16/4)	10-point-scale
7	Negreiros et al., 2020	Retrospective; University; Brazil	62 (31/31); Class I malocclusion Tx_1_: 20 self-ligating FA Tx_2_: 22 conventional FA + RME C: 20 conventional FA	Group 1: 19.4 yrs Group 2: 25.5 yrs Group 3: 21.8 yrs	Smile photo	55 laypersons (18/37) 70 orthodontists (26/44)	10-point scale
8	Reis et al., 2021	Retrospective; Private clinics; Brazil	30(13/17); Class III malocclusion Tx_1_:15; FA Tx_2_:15; OS + FA	Group1: Initial mean age: 21.26 (7.39) Final mean age: 24.52 (7.10) Group 2: Initial mean age: 23.12 (7.37) Final mean age: 25.82 (7.14)	Smile photo	44 laypeople (10/34) 67 orthodontists (27/40)	10-point scale
9	Rizzi et al. 2022	Retrospective; Private clinics; Brazil	16 (0/16) Gummy smile Tx_1_: 8 FA + MP Tx_2_: 22 OS + Le Fort I osteotomy	No specified	Smile photo	56 orthodontists (22/34) 56 Maxillo-facial Surgeons (44/12) 56 laypersons (19/37)	10-point scale
10	Thiruvenkatachari et al., 2017	Retrospective; Dental Hospital; UK	48 (16/32); Class II malocclusion Tx_1_: 14; 1CEx + FA Tx_2_: 10; 2CEx + FA C: 24; 2Ex + FA	Adolescents	Smile photo and Smiling face photo	10 laypeople 10 general dentists 10 orthodontists	10-point scale

^*^: Treatment group: Tx/Control group: C; 4Ex: 4 premolar extractions/3Ex: 3 premolar extractions/2Ex: 2 premolar extractions/1Ex: 1 premolar extractions/1CEx: 1 canine extractions/2CEx: 2 canine extractions/Non-Ex: No extractions; FA: fixed pre-adjusted appliance/RME: rapid maxillary expansion/OS: Orthognathic surgery / MP: miniplates

### Study characteristics

All the selected studies were non-randomized, eight were retrospective (Havens et al. [29]; Janson et al. [30]; Kumar et al. [31]; Meyer et al. [32]; Negreiros et al. [33]; Reis et al. [34]; Rizzi et al. [35]; Thiruvenkatachari et al. [36]) and two were purely observational (Almutairi et al. [37], Hulsey et al. [38]). The included studies were published between 1970 and 2022 in a total of eight countries. Four studies were conducted exclusively at universities (Havens et al. [29]; Janson et al. [30]; Kumar et al. [31]; Negreiros et al. [33]), four in private clinics and dental hospitals (Meyer et al. [32]; Reis et al. [34]; Rizzi et al. [35]; Thiruvenkatachari et al. [36]), one in both private clinics and university (Almutairi et al. [37]) and in one study is not specified (Hulsey et al. [38]). The descriptive characteristics of the included studies are outlined in Table 2. Two studies compared orthodontically treated patients to untreated controls (Almutairi et al. [37], Hulsey et al. [38]) and all others assessed patients' smile attractiveness after orthodontic treatment. Of all the studies, three compared pre-treatment to post-treatment smile attractiveness (Kumar et al. [31]; Reis et al. [34]; Rizzi et al. [35]). The combination of orthodontic treatment and orthognathic surgery was examined in one study (Reis et al. [34]), and another study compared orthodontic treatment to orthognathic surgery alone (Rizzi et al. [35]).

In all studies, the age of the included subjects varies from teenagers to young adults, with the exception of two studies where the age is unspecified (Kumar et al. [31], Rizzi et al. [35]). In addition, six studies included male and female study participants (Hulsey et al. [38]; Janson et al. [30]; Meyer et al. [32]; Negreiros et al. [33]; Reis et al. [34]; Thiruvenkatachari et al. [36]), three studies only included females (Almutairi et al. [37]; Havens et al. [29]; Rizzi et al. [35]) and one study did not provide information regarding sex distribution (Kumar et al. [31]). The ethnicity of the patients was mentioned only in two studies (Havens et al. [29]; Hulsey et al. [38]).

Smile attractiveness was always assessed with numbered scales and rater groups, including lay people, in all cases, except from one study that only included laypeople as raters (Hulsey et al. [38]). To minimize potential confounders, the sex distribution within all rater groups was balanced and raters were blinded regarding the outcome of the study.

### Risk of bias

**Fig. 2:**
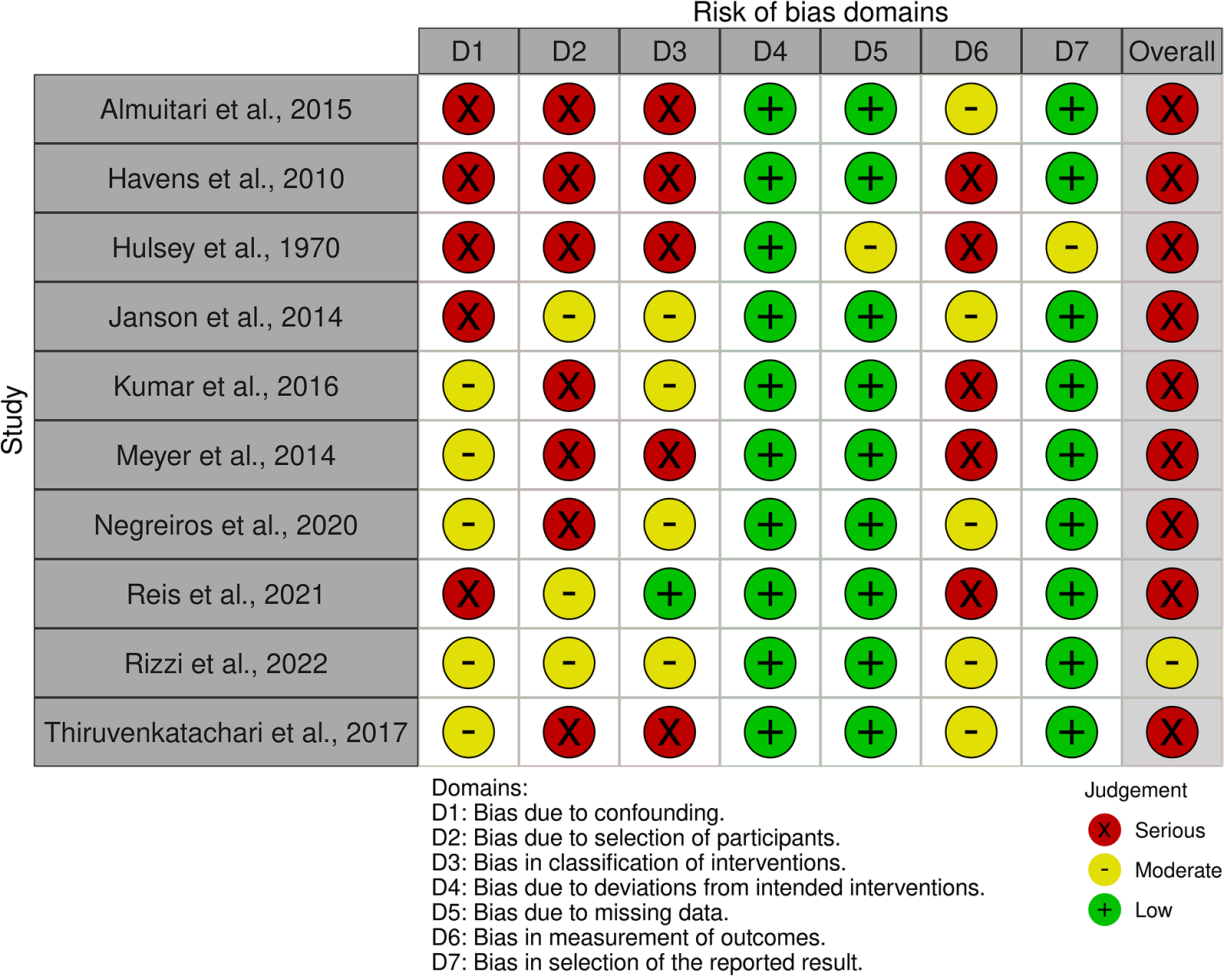
Risk of bias assessment for all included studies, as assessed with the ROBINS-I tool

**Fig. 3:**
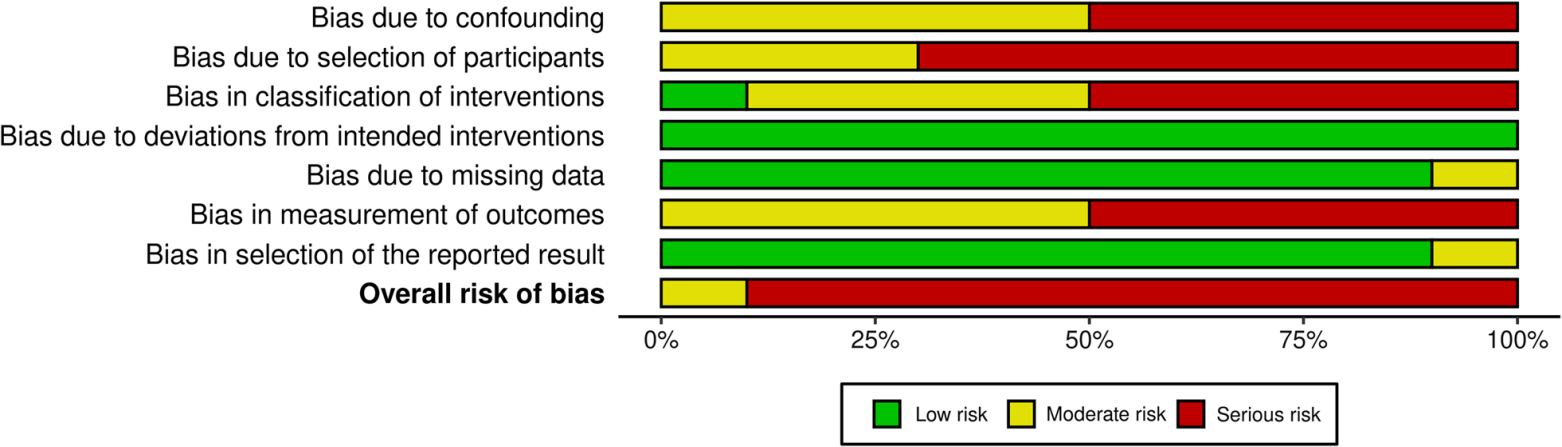
Collective data for risk of bias of all included studies, according to each domain

The risk of bias assessment using the Robins-I tool for non-randomized studies revealed that 9/10 studies presented serious risk of bias with the exception of Rizzi et al. [35] that presented moderate risk of bias. The potential sources of bias that were mostly noted upon evaluation of the studies were related to the characteristics of the samples (age and sex distribution, ethnicity and sample size), characteristics of the rater panels (age, sex and educational background), the type of records used for assessment of smile attractiveness and the applied rating scales. All studies showed a moderate to serious risk of bias in these domains. The risk of bias due to deviation from the intended intervention was low in all studies, which was expected based on their retrospective design. In two studies (Hulsey et al. [38] and Havens et al. [29]), there was no available data on the outcome assessment. All reasonable efforts were done to access these data by contacting the study authors and the libraries of the respective Universities, but they were unsuccessful. Therefore, these studies were excluded from the data synthesis. The areas of risk of bias for all 10 studies included in this review are shown in Figs. 2 and 3.

### Data synthesis

**Fig. 4:**
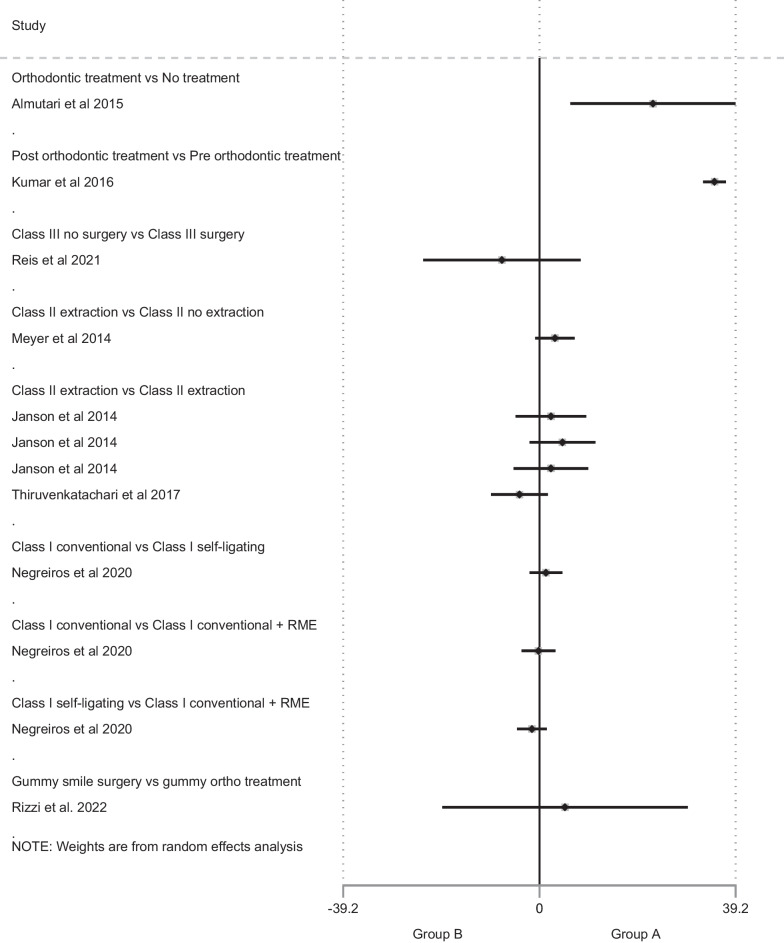
Forest plots presenting all included studies separately. Effect sizes are presented in mean differences on a scale of 0–100

Due to the large heterogeneity between the included studies in regard to the research question, study design, as well as the applied methodology, it was not possible to perform data-synthesis and a meta-analysis. However, the included studies and their individual groups (where applicable) were grouped according to intervention. In addition, all rating scales used to evaluate smile attractiveness were adjusted into a 0–100 scale to provide comparable and more easily interpretable results. The provided forest plot does not present collective results, and only displays individual study results in a graphical manner to allow for visual comparisons (Fig. 4).

## Orthodontic treatment versus no orthodontic treatment

Two studies compared smile attractiveness between patients treated orthodontically and untreated controls (Almutairi et al. [37]; Hulsey et al. [38]). Almutairi et al. compared biprotrusive patients treated with 4 premolar extractions to untreated biprotrusive subjects. The subjects were judged by different groups of raters, namely orthodontists, general dentists and laypeople. In this study, extraction of premolars resulted in a 22% improvement in smile attractiveness ratings compared to the untreated group. Interestingly, this study reported that the strictest raters were laypeople. On the contrary, Hulsey et al., who only used laypeople as assessors, reported that “orthodontically treated subjects had significantly poorer smile scores than the subjects with normal occlusion.” It can, however, not be omitted that this study was assessed of having a serious risk of bias, as was the Almutairi et al. study, but also, it did not provide any actual data. As mentioned previously, the authors of this systematic review made all reasonable efforts to retrieve these data, without success.

## Pre-orthodontic treatment vs post-orthodontic treatment

Four studies reported pre-treatment and post-treatment data (Havens et al. [29]; Kumar et al. [31]; Reis et al. [34]; Rizzi et al. [35]). Kumar et al.[31] and Havens et al.[29] studied pre- and post-treatment data of patients that had received various types of orthodontic treatment and recruited laypeople and dental professionals (orthodontists and general dentists) to assess smile attractiveness. In both studies, post-treatment smile attractiveness was rated higher than pre-treatment. The study by Kumar et al.[31] reported that smile attractiveness ratings were 35% higher after treatment. The same study also reported that laypeople gave significantly higher attractiveness scores than orthodontists and general dentists, which what not shown by Havens et al.[29]. The latter study is also the second study included in this review that did not provide any actual data in the manuscript. Attempts were made to access the data by contacting the University of Michigan as well as the first two authors; however, the data were not available.

The other two studies in this subcategory compared two different types of orthodontic treatment and presented data at two time points (pre- and post-treatment). In the study by Reis et al. 2021[34], Class III patients treated with orthodontics and surgery showed a 7.5% greater increase in smile attractiveness, compared to Class III patients treated with orthodontic camouflage. However, smile attractiveness was improved in both groups after treatment. Orthodontists’ rating scores were higher than those of laypeople, who appeared to be more critical when assessing smile attractiveness. The second study (Rizzi et al. [35]), applying a similar methodology, evaluated smile attractiveness in patients with a gummy smile treated either with orthognathic surgery (LeFort I and maxillary impaction) or with a combination of fixed orthodontic appliances and skeletal anchorage devices. Also here, post-treatment scores were overall higher than pre-treatment scores. In this study, laypeople appeared to be more accepting of a gummy smile than orthodontists and maxillo-facial surgeons.

## Orthodontic treatment A versus Orthodontic treatment B

There were six studies comparing smile attractiveness scores between different types of orthodontic treatment (Janson et al. [30]; Meyer et al. [32]; Negreiros et al. [33]; Reis et al. [34]; Rizzi et al. [35]; Thiruvenkatachari et al. [36]).

1. **Class I treatment**
  Negreiros et al. [33] compare three treatment options, namely conventional fixed appliance alone, self-ligating fixed appliance and fixed appliance combined with RME, for Class I malocclusions to assess which one improves smile attractiveness more. The study detected no statistically significant difference between the three groups.
2. **Class III treatment**
  Reis et al. [34] compared smile attractiveness in Class III patients treated with camouflage or orthognathic surgery. Although both interventions had a favorable effect on smile attractiveness, the improvement of smile attractiveness was 7.5% greater in the surgical group (*P* < 0.001). Also, orthodontists evaluated smiles as more attractive than laypeople, who were more critical, both at the beginning and at the end of treatment.
3. **Extraction treatment**
  Three studies evaluated the effect of extractions on smile attractiveness in subjects with Class II malocclusion. Meyer et al. [32] compared extraction to non-extraction cases and found no significant differences in smile attractiveness between the groups. Janson et al. [30] and Thiruvenkatachari et al. [36] studied different extraction patterns. Janson et al. [30] compared a four-premolar extraction pattern to asymmetric extraction patterns and found no differences, while Thiruvenkatachari et al. [36] also included subjects who underwent canine extractions in their study. They reported no significant differences in smile attractiveness between premolar extraction Class II cases and canine extraction Class II cases.
4. **Gummy smile treatment**
  One study (Rizzi et al. [35]), also mentioned previously, assessed the effect of surgical and non-surgical treatments on smile attractiveness and did not find any differences between them.

## Discussion

This systematic review evaluated the current literature on the effect of orthodontic treatment on smile attractiveness. An improvement in smile and facial attractiveness is the main motivating factor for patients seeking orthodontic treatment [12, 39], and therefore, there is high clinical value in answering this research question. The significance of this study is also related to the fact that it only included studies using questionnaires to rate smile attractiveness, rather than performing an assessment of smile esthetics in general. As previously mentioned, due to the substantial differences between esthetics and attractiveness [20–22], these two terms should not be used as equal when making assessments about smile or facial appearance.

Due to the large heterogeneity of the included studies in regard to their research questions, methodology and outcome assessment, a meta-analytical data synthesis was not possible. However, there are several helpful conclusions drawn by critically interpreting the individual results of the included studies. Based on the available information, orthodontic treatment improves smile attractiveness when compared to no treatment. This is based on a single investigation comparing extraction treatment to no treatment that reported a difference between groups of 22% [32]. This percentage is significantly higher when compared to the improvement that orthodontic treatment brings to overall facial attractiveness, which has been found to be approximately 9% [23]. This difference is expected since orthodontic treatment affects primarily the lower third of the face and most importantly the oral and perioral structures. A previous study evaluating the effect of anterior tooth positioning on profile shape variation found that overjet predicts 21.3% of the entire profile shape variation in a large adult population [15]. This implies that the dental configuration affects significantly the entire facial appearance, but there are also multiple other factors that exert an influence. Three-dimensional evaluations of facial appearance support this finding and have shown that facial attractiveness is related to the eyebrow ridges, the chin, the lips, the nose, as well as other factors, such as facial symmetry, facial averageness, skin tone, and eye color [40–45]. When it comes to the attractiveness of the smile, however, teeth play a much more notable role. The amount of tooth and gingival exposure, the presence of black triangles and occlusal cants have all been related to smile attractiveness [14, 46, 47], in addition to tooth-related factors, such as the dimensions of the smile and the thickness of the lips [46, 48–50]. The potential impact that orthodontic treatment has on many of those features explains the significant effect on the attractiveness of the smile detected by the present study.

Here it must be noted that an attractive smile contributes significantly to the overall attractiveness of an individual. In most cases, studies assessing facial attractiveness utilize three-dimensional or two-dimensional photographs and profile outlines of resting faces. Nevertheless, a smiling face is viewed as more attractive than a non-smiling face and elicits different neurological responses at the level of the orbitofrontal brain cortex [8, 51, 52]. In addition, the perception of a smiling face as an attractive stimulus is observed since the very early stages of life, in newborns and neonates [53]. As professional, social and personal interactions become more frequent, smiling plays an increasingly important role in life, because it elicits positive reactions and creates signals of attractiveness and trustworthiness [54–57]. Within this scope, orthodontic treatment has a positive impact in many aspects of life by significantly improving the attractiveness of the smile.

When various types of orthodontic treatments were compared, it appeared that all types of orthodontic treatment improved smile attractiveness, with no type of treatment showing a more favorable effect than others. This was stated in all relevant studies, with the exception of the study by Reis et al. [34], which included subjects with Class III malocclusions who were either treated with orthodontic camouflage or with a combination of orthodontics and orthognathic surgery. In that study it was reported that the surgical treatment improved smile attractiveness by 7.5% more than camouflage treatment. However, the authors of that study mentioned that this result is probably attributed to the fact that all included cases had moderate-to-severe malocclusions, which are better treated surgically. Taking into consideration that both treatments increased smile attractiveness by more than 24%, it is not unjustified to assume that the difference between them falls within the range of clinically acceptable variation in outcomes. Therefore, it is important to evaluate each patient individually taking into consideration the malocclusion characteristics and the patient’s treatment expectations. In cases, for example‚ where the patient declines any type of surgical intervention, choosing a camouflage treatment, that respects the biological boundaries of tooth movement, may be adequate to provide a pleasing smile appearance that meets the patient’s wishes. Similar studies that have compared surgical to non-surgical orthodontic treatments regarding their effect on facial attractiveness have shown that surgical treatments improve facial attractiveness by approximately 5% more than non-surgical ones [23]. These small differences reported by studies comparing surgical to non-surgical treatments should, however, be interpreted within the context of the examined samples. Since it would be unethical to treat severe surgical discrepancies without orthognathic surgery, it is expected that all available studies have evaluated borderline cases that could have been treated both ways. It goes without say that in patients with significant skeletal malocclusions, surgical interventions are the only ones that could offer a good occlusion and a pleasing facial appearance. In cases where patients refuse orthognathic surgery and are only willing to undergo orthodontic treatment, the benefits of the treatment are highly dependent on the possibility to achieve a functional and esthetically pleasing relationship of the dentition, especially of the anterior teeth. In the included studies, there is no information regarding subjects’ individual attitude toward surgery.

Three studies included in this systematic review assessed the association between smile attractiveness and premolar or canine extractions in Class II cases. Although the large heterogeneity between them did not allow for reasonable data synthesis, it can be concluded that none of the studies showed a significant difference between groups. This confirms what is frequently reported in the current literature, that premolar extractions do not have a negative impact on facial esthetics, in most cases [19, 58–60]. As mentioned previously, smile attractiveness is related more to anterior tooth and gingival display, to the presence of occlusal cants and to the presence of large buccal corridors [14, 46, 47]. None of these features are dependent on the presence of all premolars in the arch. If proper mechanics are applied during treatment, the position of the anterior dentition is not compromised after the extraction of premolars and thus, optimal smile esthetics can be achieved. This result is confirmed in a previous systematic review investigating the effect of orthodontic treatment on facial attractiveness, which indicated that current evidence shows no significant difference between extraction and non-extraction cases [23].

The qualitative assessment of all included studies exhibited that 9 out of 10 presented serious risk of bias and only one study (Rizzi et al. [35]) presented moderate risk of bias. The most common source of bias was the absence of pre-treatment ratings for smile attractiveness. Only 2 studies (Reis et al. [34] and Rizzi et al. [35]) included pre- and post-treatment data and thus, their results are considered more reliable than the ones from the other studies. In addition, three studies did not specify the type of malocclusion, introducing a significant confounding factor regarding the duration and the complexity of the applied treatment. Two studies (Havens et al. [29] and Kumar et al. [31]) only included one group of participants who all underwent orthodontic treatment and compared pre- and post-treatment ratings of smile attractiveness. It is speculated that, before treatment, the participants of those studies were all dissatisfied with the appearance of their smiles, therefore seeking treatment, and thus the recorded effect was probably emphasized.

Furthermore, the included studies have all used two-dimensional images for the evaluation of smile attractiveness. Seven studies used photos of the smile only (Almutairi et al. [37]; Hulsey et al. [38]; Janson et al. [30]; Kumar et al. [31]; Negreiros et al. [33]; Reis et al. [34]; Rizzi et al. [35]), two used a combination of a smiling facial photo and a photo of the smile (Havens et al. [29] and Thiruvenkatachari et al. [36]) and one study only used photos of smiling faces (Meyer et al. [32]). All photos were frontal ones, with the exception of one study, which also used ¾ photos of the smile (Almutairi et al. [37]). Due to the static nature of these images it is likely that the raters’ responses would have been different had they been exposed to more dynamic smiling images, such as short videos. The use of videos would have also provided a perception of depth and create a more realistic stimulus of a smiling expression. However, video technology is not routinely used in orthodontic diagnosis and, thus, use of photographs was implemented. An alternative could be the use of three-dimensional technology to depict the smile in three dimensions. Although 3D photography is widely used to study the face in three dimensions [40, 61, 62], when it comes to the smile its applicability is limited because the teeth are not depicted well and thus a main feature of the smile appears distorted. All but two of the included studies (Thiruvenkatachari et al. [36] and Rizzi et al. [35]) removed the colors from the images, before they were evaluated by the raters. Although this is advantageous because it removes possible distractors, it also creates an unnatural image of a smile. Skin tone and texture influence our perception of attractiveness [45, 63], which may indicate that images with color would probably lead to higher overall ratings in smile attractiveness. The results should also be interpreted taking the age of the studied subjects into consideration. All studies examined young adults or adolescents, who have notable differences in facial expressions than older adults. Due to the changes in facial soft tissues occurring with aging, the appearance of the smile is also affected, primarily due to the decrease in tooth exposure and the reduction in lip thickness [64]. All aforementioned factors contributed to the heterogeneity among studies that makes direct comparisons of the outcomes difficult.

The limitations of the available studies indicate a need for more comprehensive investigations regarding this important research topic in orthodontics. Studies with larger samples, more representative of the general population, with better sample selection and more thorough methodologies, including objective orthodontic treatment outcome assessments, would provide more reliable results and facilitate orthodontists in making more educated diagnostic decisions during treatment planning and outcome assessment.

## Conclusions

Limited evidence shows that orthodontic treatment has a moderately positive effect on the attractiveness of the smile. Based on the results of a single study, extraction treatment improves smile attractiveness by 22%, when compared to no treatment. Surgical correction of Class III cases increases smile attractiveness by 7.5% more than camouflage treatment, in cases of moderate-to-severe malocclusion. Other comparisons between various types of orthodontic treatment did not show significant differences in the amount of improvement in smile attractiveness. Nevertheless, there is significant heterogeneity and bias in the current literature assessing the effect of orthodontics on smile attractiveness, therefore the results cannot be accepted with certainty.

## Supplementary Information

**Additional file 1: Supplementary Table 1.** Electronic databases searched (first search).

**Additional file 2: Supplementary Table 2.** Reasons for exclusion of studies after reviewing the full texts against the eligibility criteria.

## Data Availability

All data are provided within the manuscript and in the supplemental material.
