# Supplementary material for: The effect of orthodontic treatment on smile attractiveness: a systematic review
Source: Prog Orthod. 2023 Feb 6;24:4. doi: 10.1186/s40510-023-00456-5 (PMC9899877; doi:10.1186/s40510-023-00456-5)
Supplement: Supplementary file 1 — Additional file 1: Supplementary Table 1. Electronic databases searched (first search). [file 40510_2023_456_MOESM1_ESM.docx]

Supplementary table 1: Electronic databases searched (first search)

| Database | Search Strategy | Limitations | Hits |
| --- | --- | --- | --- |
| MEDLINE  searched through PubMed  on 14 September, 2022  http://www.ncbi.nlm.nih.gov/pubmed/ | orthodon* AND smile [Title/Abstract] AND attractiv* [Title/Abstract] | None | 190 |
| Cochrane Database of Systematic Reviews  on 14 September, 2022 http://onlinelibrary.wiley.com/cochranelibrary/search/ | orthodon* AND smile AND attractiv* | None | 5 |
| Cochrane Database of Abstracts of Reviews of Effects  on 14 September, 2022 http://onlinelibrary.wiley.com/cochranelibrary/search/ | orthodon* AND smile AND attractiv* | None | - |
| Cochrane Central Register of Controlled Trials  on 14 September, 2022 http://onlinelibrary.wiley.com/cochranelibrary/search/ | orthodon* AND smile AND attractiv* | None | - |
| Virtual Health Library  on 14 September, 2022 http://regional.bvsalud.org/ | orthodon* AND smile AND attractiv* | None | 207 |
| Scopus  on 14 September, 2022 http://www.scopus.com/ | orthodon* AND smile AND attractiv* | None | 201 |
| Web of Science  on 14 September, 2022 https://www.webofscience.com/ | orthodon* AND smile AND attractiv* | None | 301 |
| Sum | | | 904 |
| Sum after removal of duplicates | | | 397 |
