# Supplementary material for: The effect of orthodontic treatment on smile attractiveness: a systematic review
Source: Prog Orthod. 2023 Feb 6;24:4. doi: 10.1186/s40510-023-00456-5 (PMC9899877; doi:10.1186/s40510-023-00456-5)
Supplement: Supplementary file 2 — Additional file 2: Supplementary Table 2. Reasons for exclusion of studies after reviewing the full texts against the eligibility criteria. [file 40510_2023_456_MOESM2_ESM.docx]

Supplementary Table 2. Reasons for exclusion of studies after reviewing the full texts against the eligibility criteria.

| **Nr.** | **Paper** | **Decision** |
| --- | --- | --- |
| 1 | Akyalcin S, Frels LK, English JD, Laman S. Analysis of smile esthetics in American Board of Orthodontic patients. Angle Orthod. 2014;84(3):486-91. | Excluded; assessing factors that make a smile attractive |
| 2 | Hata K, Arai K. Dimensional analyses of frontal posed smile attractiveness in Japanese female patients. Angle Orthod. 2016;86(1):127-34. | Excluded; rating esthetics based on orthodontic objective criteria |
| 3 | Kau CH, Christou T, Xie RB, Abou-Saleh T. Rating of smile attractiveness of patients finished to the American Board of Orthodontics standards. J Orofac Orthop. 2020;81(4):239-248. | Excluded; assessing factors that make a smile attractive |
| 4 | Schabel BJ, McNamara JA, Baccetti T, Franchi L, Jamieson SA. The relationship between posttreatment smile esthetics and the ABO Objective Grading System. Angle Orthod. 2008;78(4):579-84. | Excluded; rating esthetics based on orthodontic objective criteria |
| 5 | Kantharaju VH, Shivaprakash G, Shamnur N. The Relationship between Posttreatment Smile Esthetics and the ABO Objective Grading System: Class I Extraction versus Non-Extraction Cases. Turk J Orthod. 2020;34(1):39-45. | Excluded; evaluating the methods of evaluation not the smile attractiveness |
| 6 | Sarul M, Antoszewska-Smith J, Park HS. Self-perception of smile attractiveness as a reliable predictor of increased patient compliance with an orthodontist. Adv Clin Exp Med. 2019 ;28(12):1633-1638. | Excluded; evaluating the patients compliance not the smile attractiveness |
| 7 | Schabel BJ, Franchi L, Baccetti T, McNamara JA Jr. Subjective vs objective evaluations of smile esthetics. Am J Orthod Dentofacial Orthop. 2009;135(4 Suppl):S72-9. | Excluded; evaluating the methods of evaluation not the smile attractiveness |
| 8 | Ahrari F, Heravi F, Rashed R, Zarrabi MJ, Setayesh Y. Which Factors Affect Dental Esthetics and Smile Attractiveness in Orthodontically Treated Patients? J Dent (Tehran). 2015;12(7):491-503. | Excluded; assessing factors that make a smile attractive |
| 9 | Golshah A, Serenjianeh AM, Imani MM. Smile attractiveness of Persian women after orthodontic treatment. Am J Orthod Dentofacial Orthop. 2020;158(1):75-83. | Excluded; assessing factors that make a smile attractive |
| 10 | Ribas J, Paço M, Pinho T. Perception of facial esthetics by different observer groups of Class II malocclusion with mandibular retrusion. Int J Esthet Dent. 2018;13(2):208-219. | Excluded; simulation of treatment |
| 11 | Soh J, Wang ZD, Zhang WB, Kau CH. Smile Attractiveness Evaluation of Patients Selected for a U.S.-Based Board Certification Examination. Eur J Dent. 2021;15(4):630-638. | Excluded; assessing factors that make a smile attractive |
| 12 | Aline dos Santos Letieri, Clarissa Christina Avelar Fernandez, Stefanni Olga Aguiar Sales Lima, Lucianne Cople Maia, Raildo Silva Coqueiro, Matheus Melo Pithon, Age and aesthetics perception related to different types of orthodontic devices. Is there a relationship?, J World Fed Orthod, Volume 7, Issue 1, 2018, Pages 29-33 | Excluded; simulation of treatment |
| 13 | Malhotra S, Sidhu MS, Prabhakar M, Kochhar AS. Characterization of a posed smile and evaluation of facial attractiveness by panel perception and its correlation with hard and soft tissue. Orthodontics (Chic.). 2012;13(1):34-45. | Excluded; no orthodontic treatment performed |
| 14 | Schabel BJ, McNamara JA Jr, Franchi L, Baccetti T. Q-sort assessment vs visual analog scale in the evaluation of smile esthetics. Am J Orthod Dentofacial Orthop. 2009;135(4 Suppl):S61-71. | Excluded; evaluating the methods of evaluation not the smile attractiveness |
| 15 | Armalaite J, Jarutiene M, Vasiliauskas A, Sidlauskas A, Svalkauskiene V, Sidlauskas M, Skarbalius G. Smile aesthetics as perceived by dental students: a cross-sectional study. BMC Oral Health. 2018;18(1):225. | Excluded; no orthodontic treatment performed |
| 16 | Kawashima Y, Kure K, Arai K. Cephalometric characteristics of postorthodontic female patients with attractive and unattractive frontal posed smiles. Angle Orthod. 2018;88(6):797-805. | Excluded; rating esthetics based on cephalometric characteristics |
| 17 | Christou T, Abarca R, Christou V, Kau CH. Smile outcome comparison of Invisalign and traditional fixed-appliance treatment: A case-control study. Am J Orthod Dentofacial Orthop. 2020; 157(3):357-364. | Excluded; rating esthetics based on orthodontic objective criteria |
| 18 | Cheng HC, Wang YC. Effect of nonextraction and extraction orthodontic treatments on smile esthetics for different malocclusions. Am J Orthod Dentofacial Orthop. 2018;153(1):81-86. | Excluded; rating esthetics based on orthodontic objective criteria |
| 19 | Yu XN, Bai D, Feng X, Liu YH, Chen WJ, Li S, Han GL, Jiang RP, Xu TM. Correlation Between Cephalometric Measures and End-of-Treatment Facial Attractiveness. J Craniofac Surg. 2016;27(2):405-9. | Excluded; assessing facial attractiveness |
